# The Fate of Failed Debridement, Antibiotics, and Implant Retention in Infected Knee Arthroplasties: Nothing to Lose

**DOI:** 10.7759/cureus.18946

**Published:** 2021-10-21

**Authors:** Özkan Öztürk, Mahmut Özdemir, Mehmet Cenk Turgut, Murat Altay

**Affiliations:** 1 Department of Orthopedics and Traumatology, Erzurum Regional Training and Research Hospital, Erzurum, TUR; 2 Department of Orthopedics and Traumatology, VM Medical Park Hospital, Ankara, TUR; 3 Department of Orthopaedics and Traumatology, Keçiören Healthy Practice and Research Center, Ankara, TUR

**Keywords:** patient reported outcomes, prosthesis fixation, prosthesis failure, knee replacement arthroplasty, prosthesis-related infections

## Abstract

Purpose

The frequency of periprosthetic knee infections increases yearly because of the popularity of the total knee prostheses. Revision knee arthroplasty is an annoying problem for both the surgeons and the patients. Debridément, antibiotics, and implant retention (DAIR) is a popular alternative for the treatment of periprosthetic knee infections. Little is known about the fate of the failed DAIR patients. This study aims to investigate the effect of the failed DAIR on the clinical result after two-staged revision arthroplasty.

Method

Ninety-nine two-staged revision arthroplasties and 85 DAIR patients from two reference clinics were retrospectively analyzed. The minimum follow-up was 36 months. Patients were grouped according to the treatment as, two-staged revision without DAIR, two-staged revision after failed DAIR, and successful DAIR. Their Knee Society Scores (KSS), functional KSS (KSS-f) and Western Ontario and McMaster Universities Arthritis Index (WOMAC) scores were analyzed and compared.

Results

DAIR has a 52.9% success rate for the treatment of infection. Elevated erythrocyte sedimentation rates and C-reactive peptide levels are not risk factors for failure, but the time passed since the index surgery is a risk factor for worse outcome scores. Failed DAIR is not a risk factor for reinfection after two-staged revision. Last KSS after failed DAIR, successful DAIR, and two-staged revision were 83.98±7.033, 91.89±4.386, and 91.38±4.735, respectively. Last KSS-f after failed DAIR, successful DAIR, and two-staged revision were 86.25±9.524, 94.56±8.106, and 94.85±5.996, respectively. Last WOMAC after failed DAIR, successful DAIR, and two-staged revision were 86.16±7.745, 94.750±4.964, and 93.319±5.961, respectively.

Conclusion

Failed DAIR is associated with lesser, but still good, or excellent clinical scores. DAIR is suggested as a promising treatment option for periprosthetic knee infections in well-selected patients.

## Introduction

Total knee arthroplasty (TKA) is a standard surgical procedure utilized for knee arthritis, with a 1.52% prevalence in the USA [1]. With the trend of increment of incidence of TKA, revision TKA (rTKA) will also increase and be an economic burden for health care systems [2]. Infection is the most common reason for the failure of TKA, accounting for 25.2% of all the rTKAs [3].

Two-stage revision is the gold-standard treatment of periprosthetic joint infections (PJI). Still, because of the high morbidity and economic burden [2], treatment modality, which consists of debridement and antibiotics with implant retention (DAIR), is gaining popularity [4]. DAIR is recommended for early, and haematogenous infection manifested in the last three weeks [5]. The contraindications for DAIR are the inability of wound closure, the presence of sinus tracts, and loosening of the prosthesis [5]. DAIR is reported to be successful in 22.6%-83% of the patients [6]. The main reasons for failure are highly-virulent organisms, comorbidities like chronic obstructive lung disease and diabetes mellitus, age older than 85, and DAIR performed after more than 30 days of index arthroplasty [7-9].

There is plenty of literature about the success of DAIR [6], but less is reported about failed DAIR's fate. Xu et al. reported that failed DAIR is not a risk factor for reinfection after two-staged rTKA [10], and Dzaja et al. reported that failed DAIR is not related to worse outcomes after two-stage revision [11]. On the other hand, there are some reports claiming worse outcomes after failed DAIR [12].

The aim of this study is to determine whether the failed DAIR is a risk factor for reinfection and to determine the effect of the failure of DAIR on the outcome after two-stage revision.

## Materials and methods

Patients

After obtaining institutional review board approval (Erzurum Training and Research Hospital IRB Number: 2021/04-68, patients who underwent surgery for infected knee prosthesis in two referral centers between 2011 and 2017 were retrospectively analyzed. Inclusion criteria were surgical treatment of periprosthetic knee infection with DAIR or two-stage revision and the follow-up period of more than three years. Exclusion criteria were short follow-up, treatment other than DAIR or two-stage revision. Since the aim of this study is to determine the effect of failed DAIR on the outcomes after two-stage revision, two-stage revision without the history of failed DAIR is also included.

Age, sex, preoperative and postoperative complete blood counts, serum ESR and CRP values, roentgenograms, Knee Society Scores (KSS), functional Knee Society Scores (KSS-f), and Western Ontario and McMaster Universities Arthritis Index (WOMAC) were all recorded. Turkish version of KSS used in this research was shown to be reliable and valid [13]. Turkish version of WOMAC used in this research was shown to be reliable and valid [14]. Comorbidities of the patients were evaluated with age-adjusted Charlson Comorbidity Score, which is a weight-adjusted scoring system for comorbidities, including age, history of myocardial infarction, congestive heart failure, peripheral vascular disease, cerebrovascular event or transient ischemic attack, dementia, chronic obstructive lung disease, connective tissue disease, peptic ulcer, liver disease, diabetes mellitus, hemiplegia, chronic kidney disease, solid tumor, leukemia, lymphoma and AIDS [15]. The comorbidities are not evaluated separately since the index comprehends most of them. 

Eight patients who underwent knee arthrodesis because of the large bony defects were excluded. Fifteen patients were excluded from the study because of a follow-up shorter than 36 months. Five patients were excluded because of death earlier than three years. Totally 28 patients were excluded, and 184 patients were included in the study. The minimum follow-up was 36 months, and the mean follow-up was 43,8±7,62 months.

Patients were grouped according to the treatment as successful DAIR, two-stage revision after failed DAIR, and two-stage revision without DAIR.

Written informed consents of the patients for both the treatment and usage of their clinical data for scientific purposes were obtained.

Treatment protocols

Patients were diagnosed as infected TKA according to the MSIS criteria in 2011 (Table 1) [16].

**Table 1:** MSIS 2011 criteria for periprosthetic infection. One major or at least four minor criteria are accepted as periprosthetic infection. Reproduced from [16]. MSIS: Musculoskeletal Infection Society.

Major criteria	Minor criteria
A sinus tract linked with the prosthesis	Elevated serum ESR and CRP concentrations
Isolation of the same pathogen in at least two of six periprosthetic tissue or fluid cultures	Elevated synovial leucocyte count
	Elevated synovial neutrophil percentage
	Presence of purulence
	Isolation of a microorganism from one tissue or fluid culture
	More than five neutrophils per high-power field in five high-power fields of histologic analysis at 9400 magnification

**Figure 1:**
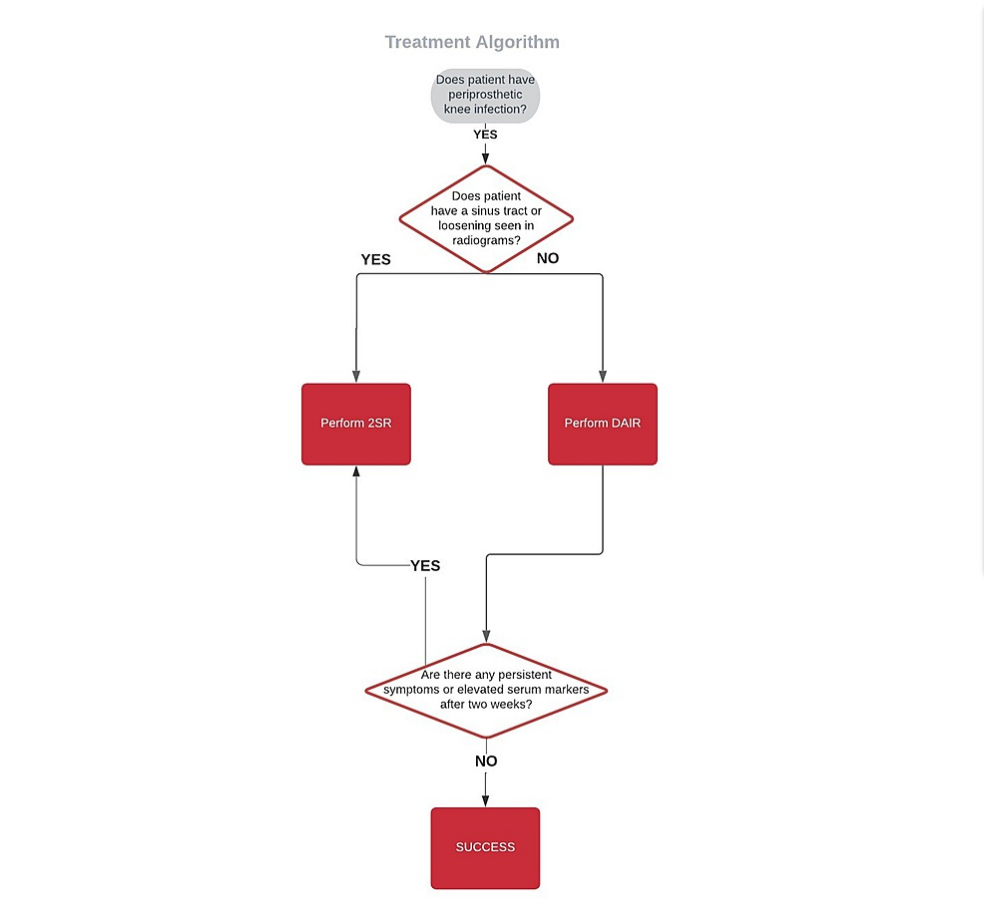
Treatment algorithm. DAIR: debridément, antibiotics and implant retention.

Standing anteroposterior and lateral radiograms were evaluated, and if periprosthetic lysis was absent and there was no fistula, DAIR was the treatment of choice. Otherwise, two-stage revision was done. If instability of the implants was detected intraoperatively, then two-stage revision was done instead of DAIR, and those five patients were included in that group. While the late infection is not a contraindication for DAIR [5], the timing of infection was not considered in the treatment strategy. Two senior surgeons conducted all the treatment choices and surgeries. The treatment algorithm is summarised in Figure 1.

All the surgeries were done under spinoepidural anesthesia. Under sterile preparations and usage of sterile tourniquets, the skin incision was made by using the previous surgical scars. The medial parapatellar approach was utilized to reach the joint. One synovial tissue sample was examined under microscopy by an expert pathologist during the surgery. Six periprosthetic tissue samples from six aspects of the joint were taken for tissue cultures. If the DAIR protocol would be utilized, then the polyethylene liner was changed. Thorough irrigation with normal saline solution and total synovectomy was performed. If two-staged revision would be done, all of the implants were carefully removed, and a gentamycin-loaded spacer was vaguely implanted. 1 gr tranexamic acid was administered via intravenous route 10 minutes before the end of all surgeries. A negative pressure drain was put in the joint, and the wound was closed appropriately.

If a two-stage revision was performed, the second-stage surgery was done after at least eight weeks. If serum CRP and ESR were still high in the eighth week, then the spacer was changed, cultures were obtained and reexamined. The second stage revision knee arthroplasty was performed with a constrained nonhinged design prosthesis (NexGen® Revision Instrumentation System; Zimmer, Warsaw, IN, USA).

After obtaining the cultures, the first dose of intravenous teicoplanin was administered intraoperatively since it is one of the recommended options in the 2013 Proceedings of the International Consensus on Periprosthetic Joint Infections [5], and easier to administer compared to vancomycin [5]. Daily 400 mg of teicoplanin was administered under an infectious disease specialist's supervision for at least eight weeks. We could not consult these patients to the same surgeon or infectious disease specialist because of the two centers in two different cities, but MSIS 2013 criteria were followed in the infectious diseases departments in both cities. If the microorganism was resistant to teicoplanin or the signs and symptoms of infection were not relieved, the antibiotic was changed under the infectious diseases specialist's guidance.

Postoperative analgesia was obtained with an epidural patient-controlled analgesia system including 0.125 mg/cc bupivacaine per 5 cc/h for 24 hours. For the following days spent inpatient, analgesia was obtained with 50 mg etodolac diluted with 100 cc saline solution via intravenous route, twice a day, and 500 mg paracetamol via intravenous route, three times a day. In chronic kidney failure patients, analgesia was obtained with intravenous paracetamol administration only. Opioids were rarely used for resistant pains under the supervision of an algologist.

Thromboprophylaxis was done with subcutaneous 40 cc enoxaparin injections daily for one month, starting 12 hours before the surgery. Mechanical thromboprophylaxis was done with medical compression stockings.

Patients were checked in weekly periods for two months, in monthly periods for one year, and yearly after one year. Relief of symptoms, normal levels of serum ESR and CRP levels, and lack of osteolysis in the radiograms were accepted as successful infection treatment.

Persistently elevated levels of serum ESR and CRP after two weeks, swelling, temper, and purulent discharge of the joint, and persistent symptoms of the patient such as swelling and continuous pain were accepted as a failure after DAIR. If serum ESR and CRP levels were elevated even mildly in the asymptomatic patients, another possible reason, like another infection or tumor, was searched. After that, these mild elevations were also accepted as failures. Two-stage revision procedure was performed on these patients, as explained above.

Statistical analysis

Statistical analyses were performed with software SPSS v24.0 (IBM Corp., Armonk, NY).

Skewness and kurtosis of the variables were calculated, and the distribution was accepted as normal if the skewness and kurtosis were between -3 and +3. Since all the continuous variables were normally distributed, the groups were compared with independent samples t-test or one-way ANOVA test. Post hoc analysis after ANOVA test was done with Tamhane’s T2 test because of the heterogeneity of the variances. Categorical variables were analyzed with the Chi-square test. Correlations were analyzed with Pearson correlation test.

## Results

Descriptives

Sixty-five of the patients (35.3%) were male, and the mean age was 69.06±6.45. The age and sex distribution and Charlson comorbidity indexes of the patients among the groups are listed in Table 2. Of the 85 patients treated with DAIR, 40 (47.05%) were reinfected and treated with two-stage revision.

**Table 2:** Age and sex distribution and Charlson comorbidity indexes among the groups. DAIR: débridement, antibiotics, and implant retention.

	Failed DAIR	Successful DAIR	Two-stage revision	Overall
Age (years)	69.70±7.321	68.47±5.837	69.07±6.397	69.06±6.458
Sex (male/female)	12/28	15/30	38/61	65/119
Charlson comorbidity index	4.25±1.629	3.11±1.210	3.23±1.26	3.42±1.40

Microorganisms

No microorganism, including atypical bacteria and fungi, was isolated in 38 (20.7%) of the patients. Methicillin-resistant Staphylococcus epidermidis (MRSE) was isolated in 41 (22.28%) of the patients, methicillin-sensitive staphylococcus epidermidis (MSSE) in 44 (23.91%), enterococcus faecalis in five (2.71%), staphylococcus lugdudensis in two (1.08%), staphylococcus lentus in one (0.54%), streptococcus gordonii in one (0.54%), Group A β-hemolytic streptococci in four (2.17%), Escherichia coli in four (2.17%), Serratia marcessens in four (2.17%), Pseudomonas aeruginosa in 12 (6.52%) and Aspergillus niger in two (1.08%) of the patients. Mixed flora was isolated in 26 (14.1%) of the patients. Gram-positive microorganisms were grouped according to the methicillin resistance, and 46 (25.0%) were methicillin-resistant while 52 (28.3%) were methicillin-sensitive. E. coli, S. marcessens, and P. aeruginosa were grouped as Gram-negative microorganisms.

Risk factors for reinfection

The two-stage revision after failed DAIR was 100% successful in terms of successful treatment of infection. Two-stage revision without previous DAIR was 98.0% successful. Only two patients in this group had an early infection after revision, which was then successfully treated with DAIR. Previous history of failed DAIR is not found to be a risk factor for reinfection (Fisher exact test, p=1.0).

Failed and successful DAIR groups were statistically compared in terms of preoperative serum ESR and CRP levels; time passed since the index surgery, time passed since the onset of the symptoms, and preoperative clinical scores (Table 3). According to this analysis, age, preoperative ESR and CRP levels do not correlate with the success of DAIR. Also, days passed since the index arthroplasty is not a significant risk factor, but days passed since the onset of symptoms is associated with failure of DAIR. Lower preoperative WOMAC and KSS-f scores were found to be associated with the failure.

**Table 3:** Several risk factors for the failure of DAIR. DAIR: débridement, antibiotics, and implant retention; ESR: erythrocyte sedimentation rate; KSS: Knee Society Score; KSS-f: functional Knee Society Score; WOMAC: Western Ontario and McMaster University Osteoarthritis Index. *Denotes significant correlation.

	Failed DAIR	Successful DAIR	p
Age (years)	69.70±7.321	68.47±5.837	0.397
Sex (male/female)	12/28	15/30	0.742
Charlson comorbidity index	4.25±1.629	3.11±1.210	0.000*
Days since index arthroplasty	109.58±105.043	72.89±71.483	0.068
Days since onset of symptoms	25.97±22.827	12.22±8.936	0.001*
Preoperative ESR (mm/h)	71.8±35.54	65.9±35.58	0.34341
Preoperative CRP (mg/dL)	9.55±8.51	9.01±8.43	0.4911
Preoperative KSS	27.95±6.869	25.67±4.487	0.078
Preoperative KSS-f	3.62±5.882	16.56±8.649	0.000*
Preoperative WOMAC	15.592±5.009	23.449±4.557	0.000*

The proliferation of methicillin-resistant Gram-positive microorganisms is a risk factor for the failure, but infection with methicillin-sensitive microorganisms is a negative risk factor for reinfection after DAIR (p=0.027). The negativity of the culture, proliferation of gram-negative microorganisms, and mixed flora are not risk factors.

Risk factors for worse outcomes

Functional and patient-reported outcomes of successful DAIR, two-stage revision after failed DAIR, and two-stage revision groups were summarised in Table 4. While all the groups had good or excellent results, the outcome after failed DAIR is significantly worse. Post hoc analysis showed that outcomes after failed DAIR is worse than successful DAIR and two-stage revision, but there is no significant difference between successful DAIR and two-stage revision.

**Table 4:** Postoperative patient-reported and functional scores. DAIR: débridement, antibiotics, and implant retention; KSS: Knee Society Score; KSS-f: functional Knee Society Score; WOMAC: Western Ontario and McMaster University Osteoarthritis Index. *Denotes significant correlation.

	Failed DAIR	Successful DAIR	Two-stage revision	p
Last KSS	83.98±7.033	91.89±4.386	91.38±4.735	0.000*
Last KSS-f	86.25±9.524	94.56±8.106	94.85±5.996	0.000*
Last WOMAC	86.16±7.745	94.750±4.964	93.319±5.961	0.000*

Sex is not related to last KSS (p=0.662), last KSS-f (p=0.693) or last WOMAC (p=0.115). Also, age, days passed since the index arthroplasty or days passed since the symptoms' onset is not related to functional outcome scores (Table 5). Higher Charlson scores and worse preoperative functional scores seem to be related to worse outcomes (Table 5).

**Table 5:** Relationship between several risk factors and outcomes Correlation coefficients and p values are listed. *Denotes significant correlation. KSS: Knee Society Score; KSS-f: functional Knee Society Score; WOMAC: Western Ontario and McMaster University Osteoarthritis Index.

	KSS	KSS-f	WOMAC
Age (years)	-0.079 (0.287)	-0.151 (0.040)*	-0.067 (0.364)
Charlson Comorbidity Index	-0.185 (0.012)*	-0.219 (0.003)*	-0.167 (0.023)*
Days since index arthroplasty	0.149 (0.142)	0.052 (0.480)	0.139 (0.060)
Days since the onset of symptoms	0.128 (0.084)	0.069 (0.350)	0.121 (0.102)
Preoperative KSS	0.104 (0.159)	0.031 (0.680)	0.164 (0.026)*
Preoperative KSS-f	0.400 (<0.001)*	0.360 (<0.001)*	0.337 (<0.001)*
Preoperative WOMAC	0343 (<0.001)*	0.207 (0.005)*	0.273 (<0.001)*

Infection with methicillin-resistant organisms is related to lower last KSS (p=0.001), last KSS-f (p=0.043) and last WOMAC scores (p=0.05).

Complications

Two (2.0%) patients in the two-stage revision group had reinfections, which were successfully treated with DAIR. Four (4%) patients in the two-stage revision group had deep vein thrombosis, successfully treated with low-molecular-weight heparin.

## Discussion

In this study, we observed that failed DAIR is not a risk factor for reinfection after two-stage revision. We also observed that age, sex, preoperative serum ESR and CRP levels, and the time passed since the index arthroplasty are not risk factors for the failure of DAIR. Poor preoperative functional scores, high Charlson comorbidity index, days passed since the onset of the symptoms, and infection with methicillin-resistant microorganisms are found to be risk factors for the failure of DAIR.

Failed DAIR seems to be associated with worse outcome scores. The other factors affecting the outcome scores are higher Charlson comorbidity index, poorer preoperative functional scores, and infection with methicillin-resistant organisms. Age, sex, days passed since the index arthroplasty and the onset of symptoms are not associated with the outcome.

DAIR is a popular method for the treatment of early periprosthetic infections. Retention of the prosthesis is attractive for both the patients and the surgeons. DAIR is also economically desirable because we do not need any expensive implants. While two-stage revision arthroplasty is still considered to be the gold standard for the treatment of periprosthetic infections [5], that procedure means two major surgeries, lots of blood loss, and other complications [17].

Several conflicting studies exist about the results of DAIR. Segawa et al. reported 88% success [18] and found that the major reason for failure is compromised immune status [18]. Chung et al. declared 100% success with arthroscopic débridement [19]. We report 53.4% success with DAIR procedures, which is consistent with the existing literature [6,18,19].

While higher-than-normal ESR and CRP levels are diagnostic criteria for infection and failure of the treatment, the levels do not seem to be related to prognosis. Only positivity matters, not the level. This finding was also reported before [20,21].

The reinfection rates after failed DAIR is controversial. Sherrell et al. reported a 34% reinfection rate after two-stage revision after failed DAIR [22]. Rajgopal et al. reported a 23.86% reinfection rate in two-stage revision after failed DAIR and 15.62% reinfection in the patients undergoing direct two-stage revision [23]. In a multicenter study, Nodzo et al. reported that failed DAIR is not a risk factor for reinfection [24]. We report 100% success after two-stage revisions following failed DAIR. We attribute the 100% success of infection eradication reported in this manuscript to our strict consistency with the MSIS criteria.

The effect of failed DAIR on the clinical outcome after two-stage revision is also a matter of debate. Rajgopal et al. reported worse functional outcomes after failed DAIR [25]. Lizaur-Utrilla et al. also reported worse functional outcomes [12]. On the other hand, in a multicenter study, it is reported that failed DAIR does not worsen the outcome after two-stage revision [25]. Dzaja et al. even reported a better outcome [11]. We found that failed DAIR worsens the results after the two-stage revision. On the other hand, none of the patients had poor clinical results, and 31 of 40 patients (77.5%) had excellent results, characterized by KSS scores higher than 80, and the others had good results, characterized by KSS scores between 69 and 80.

Methicillin-resistant microorganisms [7,23] and comorbidities [8,9] are reported to be associated with failure after DAIR, and these findings are supported by our results. On the other hand, while the time passed since the index arthroplasty is reported to be associated with poorer outcomes [9], our results do not support this. Instead, time passed since the onset of the symptoms is a risk factor.

We could not find any previous study investigating the risk factors for poorer clinical outcomes other than infection after two-stage revision following the failed DAIR. We report the first evidence for the risk factors associated with worse functional and patient-related outcomes after two-stage revision after failed DAIR.

This study has its limitations. First of all, due to the study's retrospective nature, the incompleteness of data and potential recall bias may limit the study. Secondly, even there are some studies about this issue with even 16 patients [19], a more extensive group would lead to more accurate results. Some possible risk factors like smoking and low socioeconomic status were not evaluated in the study because these are self-reported entities, and from previous experiences, we know that some patients do not report these correctly. This may be another limitation.

## Conclusions

While successful DAIR yields similar results to two-staged arthroplasty and failed DAIR is associated with worse than these but still good or excellent outcomes after two-stage revision arthroplasty, and 52.9% success of eradication of infection is a reasonable chance, we highly recommend DAIR for the periprosthetic knee infections when there was no osteolysis. Multicentered randomized prospective studies with bigger groups will lead to more accurate results.
